# The impact of school closures during the COVID-19 pandemic on reading fluency among second grade students: socioeconomic and gender perspectives

**DOI:** 10.3389/fpsyg.2024.1289145

**Published:** 2024-07-05

**Authors:** Shelley Shaul, Orly Lipka, Dana Tal-Cohen, Adi Bufman, Shahar Dotan

**Affiliations:** Edmond J. Safra Brain Research Center for the Studies of Learning Disabilities, Department of Learning Disabilities, University of Haifa, Haifa, Israel

**Keywords:** COVID-19 pandemic, reading acquisition, reading fluency, comprehension, socioeconomic status, gender differences

## Abstract

**Introduction:**

The acquisition of reading skills is a crucial milestone in early education, with formal instruction and practice playing pivotal roles. The outbreak of COVID-19 led to widespread school closures and a shift to remote learning.

**Methods:**

This study aimed to investigate the effects of school closures on reading acquisition and fluency among a large sample of second-grade children, considering socioeconomic status (SES) and gender differences. In 2019, a cohort of 2228 second-grade students from 34 schools was assessed for word reading fluency and comprehension. In 2020, during the pandemic, 765 students from a subsample of 20 original schools were re-evaluated using the same measures. The study also collected school-related data.

**Results:**

The findings from the entire sample indicated no significant differences in fluency and comprehension scores between children in the second grade in 2019 and 2020. However, a significant interaction emerged when analyzing low SES versus high SES children. Children from low SES backgrounds exhibited notably lower reading scores after a year of remote learning due to the COVID-19 outbreak. Moreover, the disparity in reading scores between low SES and high SES children nearly doubled in 2020. Gender differences were also detected.

**Discussion:**

These results underscore the impact of remote learning during the COVID-19 crisis on exacerbating gaps in reading fluency and comprehension between children from high and low SES backgrounds. The implications of these findings highlight the critical role of in-person schooling and targeted support for disadvantaged students, especially during pivotal stages of reading development.

## 1 Introduction

The global outbreak of COVID-19 in early 2020 prompted widespread school closures across many countries, including Israel, resulting in a significant shift toward remote learning (Kuhfeld et al., 2020; Lake and Dusseault, 2020; United Nations, 2020). This unprecedented situation led to changes in the educational landscape, with students adapting to shortened school days delivered through technological platforms (Hall et al., 2020; Kuhfeld et al., 2020). Moreover, parents took on a more prominent role in delivering the curriculum in many instances (Reimer et al., 2021).

A fundamental milestone in early elementary education is the acquisition of reading skills. The process of learning to read involves substantial formal instruction and practice (Stanovich and West, 1989). However, the adverse impact of COVID-19 on reading acquisition was particularly pronounced among disadvantaged children who faced unequal access to educational resources (UNESCO, 2020). This disparity is a significant concern, particularly with studies highlighting potential “Matthew Effect” dynamics during the pandemic, where existing gaps in reading ability between children from different socioeconomic backgrounds could be further exacerbated (García-Muiña et al., 2021). The “Matthew Effect” concept underscores how initial advantages can magnify disparities over time (Stanovich, 1986), which, in the context of reading, could suggest that children from lower socioeconomic status backgrounds might fall behind even more in their reading development. Furthermore, parents of elementary school children reported a reduction in learning-related activities during COVID-19 closures (Andrew et al., 2020), potentially compounding challenges for struggling readers.

The second grade is a pivotal stage where children transition from decoding-based reading strategies to more fluent and accurate reading (Chall, 1983; Bar-Kochva, 2013). Although studies on the impact of COVID-19 closures on reading have emerged, many have focused on later stages of elementary school (from 3rd grade onwards; Kuhfeld et al., 2020; Engzell et al., 2021; Kaffenberger, 2021; Relyea et al., 2023). Few large-scale studies have addressed the effects of COVID-19 school closures on reading development during earlier foundational stages (Ardington et al., 2021). The Israeli Ministry of Education’s expert panel highlighted the need to investigate and comprehend gaps arising from COVID-19, particularly in early childhood, and emphasized the importance of empirical studies based on validated tools conducted at multiple time points (Kesner Baruch et al., 2021).

This study aims to address a gap in the literature by examining reading acquisition among a substantial sample of Hebrew-speaking second-grade children—an age group that has received less attention during the early elementary years. Specifically, we investigate the trajectory of fluency development among children of diverse socioeconomic backgrounds over a year, encompassing both pre-COVID-19 conditions and the subsequent year, within the same district.

### 1.1 Reading fluency development

Reading fluency is a critical skill characterized by the ability to read with automaticity, speed, accuracy, proper expression, and appropriate phrasing (National Reading Panel (US), 2000). As reading fluency advances, the cognitive load associated with decoding decreases, allowing more cognitive resources to be allocated to comprehending the text’s meaning (Wolf and Katzir-Cohen, 2001; Perfetti, 2007; Stevens et al., 2017). The progression of oral reading fluency typically takes place between the second and third grades, persistently evolving throughout the elementary years (Chall, 1983). Early elementary oral reading fluency contributes to proficient silent reading, which becomes crucial in later elementary school (Price et al., 2016). Numerous studies across diverse languages underscore the significance of reading fluency, revealing its predictive role in reading comprehension, the ultimate goal of reading (Klauda and Guthrie, 2008; Kim et al., 2010; Stevens et al., 2017; Nevo et al., 2020).

Assessing reading fluency frequently involves measuring the accurate pronunciation of words within a restricted timeframe. For instance, the Test of Word Reading Efficiency (TOWRE) evaluates the ability to pronounce printed words both accurately and fluently, reflecting the comprehension of the read words (Torgeson et al., 1999; Fuchs et al., 2001; Good et al., 2001). Proficient automatic sight-word reading is fundamental for fluid and natural text comprehension (Miller and Schwanenflugel, 2008; Kuhn et al., 2010). Thus, tests gaging the number of correctly read words within a given duration serve as valuable tools for identifying potential reading difficulties (Valencia et al., 2010). Research underscores that during early grades, reading fluency significantly contributes to comprehension, a principle that is particularly pronounced in second-grade readers (Fuchs et al., 2001; Valencia et al., 2010). Reading in context demands the activation of semantics, as readers simultaneously process words while aiming to extract textual meaning (Katzir et al., 2006). Consequently, the amalgamation of syntactic rules and semantic structures is essential for constructing cohesive units of ideas. Insufficient automation at lower processing levels (letters or words) could impede processing at higher levels (sentences or texts; Logan, 1997).

This study’s focus is on Hebrew-speaking children, with Hebrew characterized as an Abjad writing system. An Abjad writing system predominantly consists of consonantal representation with sporadic and incomplete vowel representation (Eviatar and Share, 2013). Hebrew is available in two forms: pointed (shallow orthography) and unpointed (deep orthography). Early reading acquisition in first grade revolves around shallow pointed Hebrew, allowing for rapid association between letters and sounds due to comprehensive phonological cues (Share and Levin, 1999; Shany et al., 2012). As such, most children become skilled decoders by the end of first grade, heightening the importance of speed and fluency (Lipka et al., 2016). The progression to partially pointed texts, particularly in second and third grades, exposes readers to lexico-morpho-orthographic knowledge utilization (Shany et al., 2012).

In nurturing reading fluency in first and second graders, the recommendation is for students to engage in daily reading aloud and silent practice, utilizing materials tailored to their level of competence (National Reading Panel (US), 2000; The Israeli Ministry of Education, 2014). The shift to remote instruction is believed to have potentially hindered teachers’ ability to facilitate ample reading fluency practice opportunities.

### 1.2 The challenges of remotely teaching literacy to diverse learners

The abrupt shift to remote learning during the pandemic posed significant challenges for educators, particularly in teaching literacy to young children. These learners, who had not yet become independent readers, faced obstacles in navigating technological tools independently (Sucena et al., 2022). As literacy development heavily relies on face-to-face interaction, the transition to remote learning presented hurdles in providing the necessary constant feedback and personalized attention required for learning to read and write (Relyea et al., 2023).

Teachers were thrust into an unfamiliar landscape, requiring them to adapt and innovate in the realm of online instruction with limited prior experience. This shift was especially arduous for educators in the early elementary grades (Giovannella et al., 2020; Kruszewska et al., 2020; Letzel et al., 2020; Dotan et al., 2021). A study in Israel conducted by Dotan et al. (2021) among first- and second-grade teachers revealed their struggles in remote teaching, including challenges in fostering reading fluency and comprehension, addressing the needs of struggling readers, and assessing literacy skills remotely. Beyond curriculum adaptation, teachers also encountered difficulties in teaching diverse learners. Notably, the digital divide was exacerbated by socioeconomic status (SES) disparities, with 75% of low-SES school teachers reporting unequal access to computers among their students, compared to 46% in middle-high SES schools (Dotan et al., 2021).

Despite the hurdles, some positive outcomes were observed due to school closures. The increased involvement of parents in providing home support during remote learning potentially contributed to emotional and academic advancements (Immerfall, 2020). Nonetheless, the prevailing sentiment from research indicates learning loss resulting from school absences (Kuhfeld et al., 2020; Engzell et al., 2021; OECD, 2023).

In evaluating the pandemic’s impact on learning, the term “unfinished learning” becomes relevant—a concept encompassing missed instruction due to school closures (Lambert and Sassone, 2020; The National Authority for Measurement and Evaluation in Education, 2023). Notably, this term does not imply a permanent deficit; instead, with proper support, students can attain the necessary mastery.

Additionally, the term “vulnerable children” takes on significance in this context, especially concerning children from low SES backgrounds. Their vulnerability extends to economic hardships, limited access to resources, reduced support, and heightened stress at home (Drane et al., 2020; Masters et al., 2020). The literature review reinforces the imperative to attend to these vulnerable learners, particularly those from low SES backgrounds who are at risk of accumulating academic gaps, especially in reading, during the COVID-19 period (Kaffenberger, 2021; Relyea et al., 2023).

In conclusion, the challenges of remotely teaching literacy to diverse learners during the pandemic were multifaceted. Teachers navigated the complexities of adapting to online instruction, while students faced barriers in receiving the personalized attention necessary for literacy development. The unequal access to technology further exacerbated disparities, with vulnerable learners from low SES backgrounds at greater risk of falling behind. Despite the potential benefits of home support, learning loss remained a prevalent concern. The educational community’s focus on addressing these challenges is essential for fostering equitable learning outcomes and supporting vulnerable children’s academic growth.

Several studies have attempted to estimate the extent of learning gaps resulting from school closures, drawing insights from previous instances of learning loss during periods like summer vacations or crises. Bao et al. (2020) predicted that kindergarten children in the United States would experience an average loss of 31% in their reading ability gained in 2020. Kuhfeld et al. (2020) expanded on this by demonstrating that third- to seventh-grade students could lose around 35% of their reading gains during the COVID-19 period compared to a typical school year. Furthermore, the impact was more pronounced among students with low socioeconomic status (SES). In their predictions about school achievement variability during the pandemic, they estimated a reading score decrease of 1.2 times lower than typical year scores (Kuhfeld et al., 2020). Hevia et al. (2022) examined 10-15-year-old readers and indicated that the younger readers, as well as those with low SES, showed the greatest learning loss in reading during the COVID-19 pandemic.

An interesting recent meta-analysis review (Betthäuser et al., 2023) identified 42 studies from 15 countries on learning progress among primary and secondary school children during the COVID-19 pandemic. It was found that students experienced a loss of approximately 35% of a school year’s learning. On average, the learning advancement of school-aged children was significantly reduced during the pandemic. Furthermore, the review implies that the pandemic has intensified educational disparities among children from diverse SES, which have been found before the pandemic.

This trend receives support from research on regular periods, such as the summer vacation, during which the learning loss of children from low socioeconomic backgrounds is significantly more substantial than that of those from moderate to high socioeconomic backgrounds (e.g., Burkam et al., 2004; Downey et al., 2004; Kim and White, 2008; Allington et al., 2010).

A simulation study conducted across seven low- and middle-income countries by Kaffenberger (2021) projected that a school closure lasting one-third of a regular year during third grade could lead to a year-long loss in learning until tenth grade, disproportionately affecting students in lower-income countries.

These trends have been found not only in reading but also in mathematical abilities, Blaskó et al. (2022) sought to assess the potential impact of pandemic-related learning losses in mathematics across 22 European countries, surveying 4,400 4th graders. Their study was based on data from an international achievement survey conducted before the pandemic, namely the Trends in International Mathematics and Science Study 2019. The findings revealed significant disparities among European countries regarding the availability of essential distance-learning resources, parental backgrounds, and school differences. These discrepancies in country standings are likely attributed to both the affluence of and inequalities within the respective countries, which, in turn, can impact the effect of learning loss.

A recent study conducted in the US by Relyea et al. (2023) found that the average reading achievement gain during the 2020–2021 school year was lower compared to the 2018–2019 school year. The observed effect sizes for learning loss were 0.54, 0.27, and 0.28 standard deviations for grades 3, 4, and 5, respectively. Similar gaps in reading skills were detected among second-grade students in South Africa (Ardington et al., 2021). This study compared reading skills of students assessed before (2019) and during the pandemic (2020), revealing a reading gap ranging from 57 to 70% for English-speaking second graders.

A study focused on fifth-grade students in Germany, employing real-time assessments through a reading comprehension task in 2020 after school closures, highlighted a learning loss of 11–17% compared to previous measurements (Schult et al., 2022).

A recent systematic review (Panagouli et al., 2021), synthesizing data from 42 studies primarily conducted in Europe, Asia, and America, investigates the impact of online learning and modified educational methods on school-aged students during the COVID-19 pandemic. The review encompasses students aged 8 to 22 and revealed varied effects: The most prominent trend indicated that students experienced learning loss, especially in math and reading, though some benefited. Younger students and those with neurodevelopmental disorders or special education needs faced greater challenges. Additionally, parents reported similar trends, observing declines in their children’s performance, though some noted benefits from online learning. Teachers mainly reported academic gaps, particularly in mathematics and reading. Despite challenges, younger students showed enthusiasm for interactive learning materials, suggesting their positive effects should be considered.

Furthermore, a meta-analysis of 18 studies (König and Frey, 2022) mainly from the United States and Europe (predominantly Germany and the Netherlands), assessed the impact of COVID-19-related school closures on student achievement. The analysis showed a negative effect, with a weekly learning loss of −0.022. It also tentatively suggested that younger primary school students were more adversely affected compared to older students, possibly due to their lower self-regulated learning capabilities and the vital role of teacher scaffolding in regular instruction. The analysis suggested that remote learning was more effective in later lockdown phases than initially, possibly due to the familiarity gained with established online learning apps.

A study spanning from third to ninth grade in Switzerland investigated the impact of COVID-19-related school closures and the effectiveness of in-person versus distance learning in math and language (Tomasik et al., 2021). It was found that while older students could somewhat offset the effects of school closures, younger students faced significant challenges. Learning progress for younger children not only slowed down, potentially affecting future development, but also became more varied. While a small group of primary school students benefited from closures, others experienced severe declines in performance. These children are at risk of falling behind academically, emphasizing the importance of addressing their needs.

These studies collectively underscore the pervasive impact of COVID-19-induced school closures on students’ reading skills, transcending socioeconomic, cultural, and linguistic boundaries. Overall, these findings emphasize that the pandemic’s repercussions on reading development have been particularly detrimental for children from low-SES backgrounds. Consequently, students returned to school with substantial and divergent learning gaps, necessitating targeted efforts from educators to address and mitigate these disparities. Notably, learning losses were more pronounced among students from less educated and low SES households (Engzell et al., 2021; Kaffenberger, 2021; Betthäuser et al., 2023; Relyea et al., 2023).

### 1.3 Reading and gender

Gender constitutes another significant contextual factor within the realm of children’s reading development. Despite standardized literacy instruction in classrooms, disparities in reading achievement between boys and girls have been consistently observed. Numerous studies have consistently highlighted noteworthy gender differences in reading achievement across the entire spectrum of reading abilities within educational settings (Chatterji, 2006; Mullis et al., 2007; Logan and Johnston, 2010; Robinson and Lubienski, 2011; Reardon et al., 2019).

Remarkably, girls consistently outperform boys in reading achievement (Chatterji, 2006; Mullis et al., 2007; Logan and Johnston, 2010; Robinson and Lubienski, 2011; Katzir et al., 2018; Reardon et al., 2019), and these gender differences do not display a marked declining trend across elementary or secondary schooling (Reardon et al., 2019; Reilly et al., 2019). Additionally, substantial gender imbalances exist in poor reading, with boys being disproportionately represented (Reilly et al., 2019). Notably, prior empirical evidence (Coles and Hall, 2002; Mullis et al., 2007) consistently indicates that girls report higher reading frequency compared to boys. Gender-linked disparities in reading frequency may indeed influence variations in reading performance.

Support for gender differences can be found in the latest PISA report, in which girls outperformed boys in reading by an average of 24 points across OECD countries, indicating a universal gender gap. Among low performers, boys outnumbered girls, constituting 31% compared to 22% in reading proficiency. Conversely, among top performers, girls slightly outnumbered boys, with 8% versus 6% on average across OECD nations. In Israel, ranked 30th out of 81 countries, girls achieved a mean reading score of 486, surpassing boys by 24 points (462). While girls’ literacy achievements declined compared to previous years, boys showed improvement. Despite this narrowing trend, the gender gap still favors girls in reading proficiency. The gender gap scenario in Israel closely mirrors the OECD average. The Israeli Ministry of Education emphasized, based on the PISA 2022 findings, that the gender gaps in reading proficiency translate to nearly a year of schooling.

While gender effects in remote learning have primarily been explored among older students, limited research has delved into gender-specific effects on young learners during the COVID-19 pandemic. Some studies suggest that females tend to exhibit greater adaptability to collaborative and technology-based instruction, while others find that males often display a higher comfort level with the technical aspects of remote learning platforms (Jones et al., 2021).

It is vital to underscore that most existing studies have focused on older children rather than those in the early stages of elementary school, where reading acquisition begins. As such, this present study emphasizes reading acquisition among second-grade students, aiming to bridge a gap in the literature pertaining to reading development during COVID-19. This research particularly targets children from diverse backgrounds at this pivotal stage. Furthermore, the study’s focus extends to examining whether gender-related differences manifest differently among boys and girls.

Research Questions:

1. What is the effect of COVID-19 on second-grade children’s reading fluency, and is there an interaction between COVID-19, SES, and gender on reading fluency?
2. What is the effect of COVID-19 on second-grade children’s comprehension fluency, and is there an interaction between COVID-19, SES, and gender on comprehension fluency?

## 2 Methods

### 2.1 Participants

The study included primary school students from the Israeli public education system, all Hebrew speaking children with typical IQs, encompassing various socioeconomic status (SES) backgrounds in the southern region of Israel. The participants’ age range was between seven and 8 years old, with a relatively equal distribution of boys (49%) and girls (51%). None of the children in the sample exhibited significant neurological difficulties. The division of children into SES groups was based on the Ministry of Education’s scoring system for schools, utilizing neighborhood and parental demographic information including education and income. A total of 20 schools were examined at both time points with 5% of the schools representing high SES, 55% medium SES and 40% of the schools from low SES. A comprehensive overview of sociodemographic characteristics is presented in Table 1.

**Table 1:** Sociodemographic characteristics of the sample.

Baseline characteristic	Year 1	Year 2
Gender		
Male (%)	50.1	49.1
Female (%)	49.9	50.9
Number of schools	*n* = 20	*n* = 20
School SES		
Low (%)	40	40
Medium (%)	55	55
High (%)	5	5
Percent of children in the different SES		
Low (%)	34.3	34.2
Medium (%)	59.7	62.2
High (%)	6	3.6

### 2.2 Measures

#### 2.2.1 Reading fluency

Word reading fluency was assessed using the TOWRE test (Katzir et al., 2012, based on Torgeson et al., 1999). Administered individually, participants were tasked with orally reading 80 single words as swiftly and accurately as possible within a 45-s timeframe. The words were progressively ordered in terms of complexity. Scores were computed based on the number of correct words read in 45 s and the error percentage. The internal consistency reliability (α) of this assessment was 0.95.

#### 2.2.2 Comprehension fluency

A group-administered task was employed to evaluate semantic comprehension fluency (Yinon and Shaul, 2017, based on Hutzler and Wimmer, 2004). This task consisted of 21 sentences spanning a range of everyday topics. Participants were required to read each sentence and promptly indicate whether it was semantically accurate or erroneous, all within a two-minute timeframe. The scores were calculated based on the number of accurately marked sentences within 2 min and the error percentage. The internal consistency reliability (α) for this task was 0.93.

### 2.3 Procedure

The necessary approvals were secured from the Ministry of Education and the relevant university’s ethics committee prior to data collection. All assessments were individually administered to participants in a designated quiet room within the school premises. Each assessment session lasted approximately 10 min. During the initial year of the study (October 2019), 1,460 children from 20 schools underwent testing. In the subsequent year (October 2020), 815 children were tested from the same 20 schools. All assessments were conducted individually during school hours in a controlled environment.

## 3 Results

### 3.1 First research question: the effect of COVID-19, SES and gender on reading fluency

To answer the first research question regarding the combined effect of COVID-19, SES, and gender on reading fluency, a univariate analysis of covariance (ANCOVA) was run with COVID-19, SES, and gender as independent variables, reading fluency as the dependent variable, and school as a covariate variable. The descriptive statistics of the word reading fluency is presented in Table 2. The analysis revealed no main effect of COVID-19 or gender, F’s < 1. The main effect of SES was significant, *F*(2, 1988) = 39.15, *p* < 0.001, η^2^ = 0.04, indicating that participants in the Low SES schools (*m* = 21.75, SE = 0.45) had lower reading fluency compared to medium SES (*m* = 25.67, SD = 0.35; *p* < 0.001) which were lower than the High SES (*m* = 31.64, SE = 1.32; *p* < 0.05). There were significant differences between all the different SES in reading fluency (*p* < 0.001).

**Table 2:** Mean and (SD) of word reading fluency in the among the different SES groups and gender in both years of the study.

	Year 1 2019 before COVID				Year 2 2020 after COVID			
	High SES	Medium SES	Low SES	Total	High SES	Medium SES	Low SES	Total
Boys	–	25.89 (11.31)	23.58 (11.26)	25.08 (11.33)	–	26.15 (10.33)	20.67 (10.77)	23.80 (10.85)
Girls	29.99 (10.22)	24.11 (10.32)	21.70 (9.98)	24.04 (10.48)	33.30 (13.60)	26.54 (11.13)	21.07 (8.67)	25.26 (11.09)
Total	29.99 (10.22)	25.08 (10.90)	22.66 (10.69)	24.56 (10.93)	33.30 (13.60)	26.35 (10.74)	20.84 (9.88)	24.55 (10.99)

**Figure 1:**
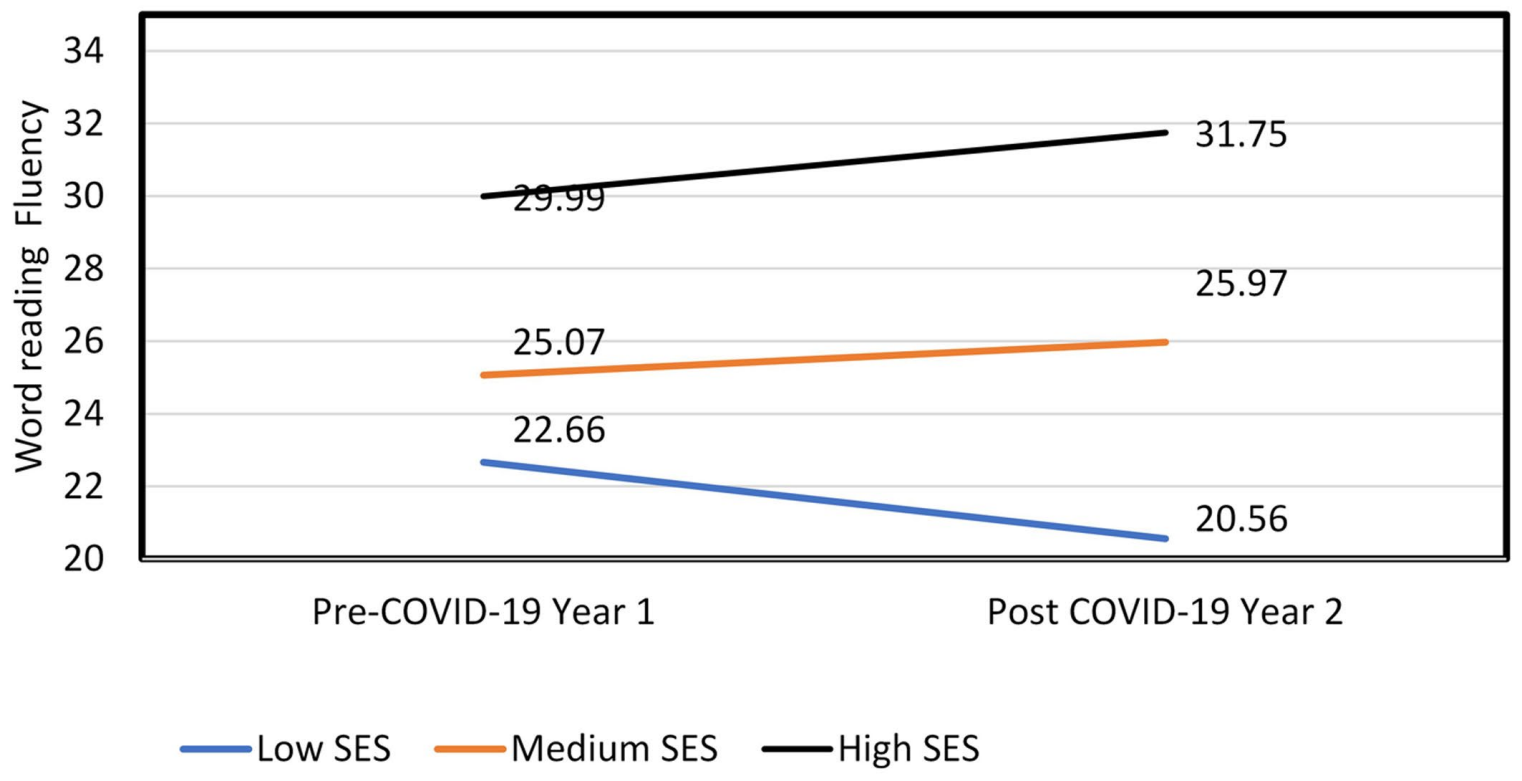
Word-reading fluency among the different SES levels in both years.

The interaction between COVID-19 and SES was significant, F(2, 1988) = 3.99, *p* < 0.05, η^2^ = 0.01. *Post-hoc* analyses revealed that the negative effect of COVID-19 existed only in low SES schools, *F*(1, 761) = 6.89, *p* < 0.01, η^2^ = 0.01. Low SES Participants in year 2 (post-COVID-19) had lower reading fluency (*m* = 20.56, SD = 0.64) than year 1 participants (pre-COVID-19; *m* = 22.66, SD = 0.47). There was no effect of COVID-19 on medium SES, *F*(1, 1,371) = 2.14, *p* = 0.14, nor High SES (*F* < 1). See Figure 1.

In addition, the interaction between COVID-19 and gender was significant, *F*(1, 1988) = 3.82, *p* = 0.05, η^2^ = 0.00. Post-hoc analyses revealed a marginally significant effect of gender on reading fluency in year 1, in year 1, *F*(1, 1,455) = 3.36, *p* = 0.07, η^2^ = 0.00, indicating that females’ reading fluency (*m* = 24.04) was slightly lower than that of males’ (*m* = 25.08, SD = 10.48). In year 2, there the performance of females was higher than the males.

The interaction between SES and gender, as well as the triple interaction between COVID-19, SES, and gender, were insignificant (F’s < 1).

Following this ANCOVA analysis, another ANCOVA analysis was run without school as a covariate variable. This analysis yielded similar trends: a significant main effect of SES, *F*(2, 1989) = 24.54, *p* < 0.001, η^2^ = 0.02, and interaction of COVID-19 and SES, F(2, 1989) = 3.99, p < 0.01, η^2^ = 0.01; a marginally significant interaction between COVID-19 and gender, *F*(1, 1989) = 3.82, *p* = 0.05, η^2^ = 0.00; and the insignificant effects were the main effects of gender and COVID-19, and the interactions of SES × COVID-19, and SES × COVID-19 × gender (all F’s < 1).

### 3.2 Second research question: the effect of COVID-19, SES and gender comprehension fluency

**Figure 2:**
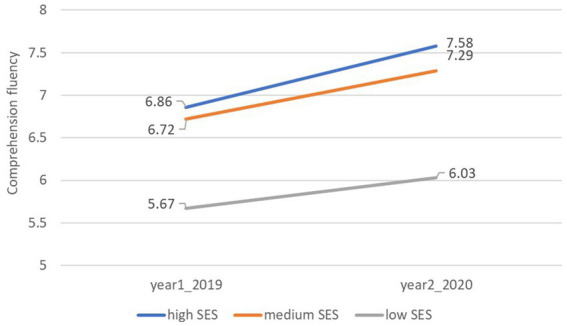
Comprehension fluency among the different SES levels in both years.

To address the second research question concerning the combined impact of COVID-19, SES, and gender on comprehension fluency, two similar univariate analyses of covariance (ANCOVAs) were conducted with COVID-19, SES, and gender as independent variables, comprehension fluency as the dependent variable, and with and without school as a covariate variable. The descriptive statistics of the reading comprehension fluency is presented in Table 3. The analysis that included school as a covariate variable revealed a significant main effect of SES, *F*(2, 1958) = 14.46, *p* < 0.001, η^2^ = 0.02, indicating that participants in low SES schools (*m* = 5.82, SE = 0.17) had lower reading fluency compared to medium SES (*m* = 7.00, SD = 0.13; *p* < 0.001) and high SES (*m* = 7.21 SD = 0.51). There were no differences in comprehension fluency between high SES and medium (*p* = 0.66) (Figure 2). This analysis did not indicate main effects of COVID-19, *F*(1, 1958) = 2.58, *p* = 0.11, or gender, *F* < 1. An examination of the interactions indicated that all interactions were insignificant: COVID-19 × gender, F(1, 1958) = 1.87, *p* = 0.17; and COVID-19 × SES, gender × SES, and COVID-19 × gender × SES, all F’s < 1.

**Table 3:** Mean and (SD) of reading comprehension fluency in the among the different SES groups and gender in both years of the study.

	Year 1 2019 before COVID				Year 2 2020 after COVID			
	High SES	Medium SES	Low SES	Total	High SES	Medium SES	Low SES	Total
Boys	–	6.90 (4.01)	5.88 (3.97)	6.55 (4.03)	–	7.16 (4.64)	5.96 (4.56)	6.65 (4.56)
Girls	6.86 (2.99)	6.49 (3.63)	5.46 (3.53)	6.20 (3.55)	7.58 (3.69)	7.4 (5.03)	6.11 (4.27)	7.01 (4.74)
Total	6.86 (2.99)	6.72 (3.84)	5.67 (3.77)	6.19 (3.56)	7.58 (3.69)	7.29 (4.84)	6.03 (4.32)	6.84 (4.65)

The ANCOVA analysis that was run without school as a covariate variable yielded similar trends: a significant main effect of SES, *F*(2, 1959) = 13.71, *p* < 0.001, η^2^ = 0.01. All other effects were insignificant: the main effects of COVID-19 m, *F*(1,1959) = 2.51, *p* = 0.11, and gender F < 1, and the interactions of COVID-19 x gender, F(1,1959) = 2.02, *p* = 0.16, SES × COVID-19, SES X gender, and SES × COVID-19 × gender (all F’s < 1).

## 4 Discussion

The acquisition of reading skills stands as a crucial milestone in early elementary education, a complex process that requires significant hours of formal teaching and practice (Stanovich and West, 1989). Against this backdrop, this study aimed to scrutinize the impact of Coronavirus-related school closures on the development of reading fluency and comprehension among second-grade students. Additionally, it aimed to assess the differential impact of COVID-19 on reading skills among second-grade students with varying socioeconomic backgrounds and to explore potential gender differences. This research was spurred by the dearth of comprehensive large-scale studies employing validated reading assessment tools across distinct time periods among children of the same age (Kesner Baruch et al., 2021). The examination of students from the same schools across both pre-pandemic and face-to-face learning periods allowed for a robust evaluation of the gaps in reading acquisition during the COVID-19 era among second-grade learners.

This study explored the influence of COVID-19 on reading and comprehension fluency in second-grade children. The assessment utilized measures of reading fluency for single words (TOWRE; Katzir et al., 2012, based on Torgeson et al., 1999) and comprehension fluency at the sentence level (semantics; Yinon and Shaul, 2017, based on Hutzler and Wimmer, 2004) in two distinct time frames among the second-grade cohort. The measurements occurred both before the onset of Coronavirus-related closures and after their resumption of face-to-face learning. Notably, the two groups of students were drawn from the same schools, exposed to the same educators and curriculum, with the sample adjusted for varying SES levels.

Surprisingly, the results demonstrated no significant disparities in reading fluency between second-grade students assessed before the pandemic in 2019 and those evaluated after the closures in 2020. A plausible explanation for the absence of discrepancies in fluency between these periods pertains to the characteristics of Hebrew orthography. The initial phases of reading acquisition in first grade encompass learning shallow pointed Hebrew, which facilitates the rapid assimilation of the correspondence between letters and sounds due to the provision of comprehensive phonological information (Share and Levin, 1999). As a result, most children become proficient decoders by the end of first grade (Lipka et al., 2016). Crucially, the two cohorts of second-grade students in this study had already acquired these foundational decoding skills during their first-grade year, preceding the pandemic’s advent. This suggests that while remote learning took place during their second-grade year, it did not notably impact the overall fluency and comprehension of these second graders as a whole.

When examining the SES effect, which focused on the differential effects of COVID-19 on reading among second-grade students of varied socioeconomic backgrounds, the study unearthed a significant SES impact on both word-reading fluency and comprehension at the sentence level. The findings highlighted that lower SES corresponded to lower reading and comprehension fluency. Moreover, a noteworthy interaction emerged specifically for reading fluency, rather than comprehension fluency, among students from diverse SES backgrounds. This interaction stemmed from a considerable decline in word-reading fluency and comprehension fluency within children from low SES during the pandemic, in contrast to their higher SES counterparts.

This decline is notable given the widely established SES-based disparities in reading fluency and comprehension (Burkam et al., 2004; Christodoulou et al., 2017). The pandemic exacerbated these gaps, revealing that children from low SES backgrounds faced substantial challenges during remote learning, potentially due to limited access to digital resources, reduced parental support, and heightened familial stress. The substantial decrease in reading fluency and comprehension abilities among low-SES children underscores the urgent need for targeted interventions to mitigate the amplified disparities brought about by the pandemic.

To conclude, the study contributes to our understanding of the ramifications of COVID-19-induced school closures on reading acquisition. The investigation suggests that the impact on reading skills might be mediated by prior decoding proficiency and underlines the significance of mitigating socioeconomic disparities. The findings underscore the urgency of tailored educational support to bridge the gaps that have emerged during the pandemic, particularly among students from low-SES backgrounds.

The observed widening gap in reading fluency and comprehension between children of low SES and those of medium-high SES during 2020 underscores a significant concern within the educational landscape (Burkam et al., 2004; Christodoulou et al., 2017). This finding highlights a pressing need for understanding the factors contributing to this phenomenon in the context of the COVID-19 pandemic. Several plausible explanations for this widening disparity emerge from the current study’s findings.

One conceivable explanation for the increased gap is rooted in the altered learning environment precipitated by school closures due to the pandemic. The significant reduction in the school day’s duration, coupled with the reliance on digital learning platforms for curriculum delivery, has had varying consequences for different student populations (Hall et al., 2020; Kuhfeld et al., 2020). Notably, the majority of second-grade children lack the autonomy required for effective engagement with digital tools, necessitating greater parental involvement. However, parents from low SES backgrounds, who might face financial concerns and time constraints, may have struggled to provide the necessary support for their children’s remote learning (Giovannella et al., 2020; Kruszewska et al., 2020; Letzel et al., 2020). This lack of adequate support could potentially contribute to the observed widening gap.

Furthermore, households with low SES often face challenges related to digital access and availability (UNESCO, 2020). Reports from teachers in low-SES schools corroborate this, revealing that many students lacked access to computers during remote learning (Dotan et al., 2021). This digital divide could have amplified the gap in reading fluency and comprehension skills, as students without access to digital tools were likely further marginalized during remote learning.

The confluence of these factors, coupled with the abrupt transition to remote learning, might have compounded the challenges faced by students from low SES backgrounds. This combined effect likely contributed to the significant decline in reading fluency and comprehension abilities among these students. This explanation finds reinforcement in a study by Domingue et al. (2021) that revealed the impact of SES on oral reading fluency growth during the COVID-19 period, where low SES students experienced a decline compared to the previous year.

Interestingly, during the pandemic, reading comprehension fluency improved among children of medium-high SES. This could be attributed to the comprehensive support these students received at home, allowing them to capitalize on one-on-one learning opportunities with parents or older siblings. This observation emphasizes the advantages of tailored support in affluent households.

In addition, while no significant gender differences were found in general, an unexpected effect of the pandemic was observed on boys. Previous literature has highlighted gendered experiences in education, with girls often encouraged more to read and boys receiving more opportunities for computing (Eccles et al., 1993). The pandemic-induced shift to remote learning could have impacted boys’ confidence and interest in computing-related learning, thereby affecting their academic performance. Conversely, the superior reading proficiency exhibited by girls on average (Logan and Johnston, 2010) and their affinity for reading could have helped them adapt better to self-regulated, computer-based learning.

The findings underscore the significance of addressing the “Matthew effect” (Stanovich, 1986) in the context of the pandemic-induced disparities. The trajectory of reading skill development may exacerbate differences over time, warranting strategic efforts to narrow these gaps. It is crucial to consider the varied impact of remote learning on different student populations and their unique challenges.

This study has several limitations, although there was a large diverse sample from different SES there were no boys in the high SES group and therefore gender differences were examined only in the medium and low SES groups. In addition, all the children were Hebrew speaking children thus the effect of school closure was not examined among bilingual children or children from different minorities, future studies should examine the long-term effect of the COVID and school closure among different types of population, and at various ages to examine the effect at different stages of reading. Furthermore, only one aspect of comprehension was examined which may limit our understanding of the effect of COVID and school closure, this topic should be further examined as well.

In conclusion, the study highlights the importance of targeted interventions to address the widening gaps exacerbated by the pandemic, particularly among students from low SES backgrounds, as well as gender differences. The repercussions of learning loss and increased stress and anxiety during the pandemic cannot be ignored. Educators and policymakers must channel resources and efforts toward supporting these vulnerable populations to ensure equitable academic outcomes. An exploration of the pandemic’s impact on diverse populations will be integral to comprehending its full educational implications.

## Data availability statement

The raw data supporting the conclusions of this article will be made available by the authors, without undue reservation.

## Ethics statement

The studies involving humans were approved by Ethics Committee University of Haifa Faculty of Education Chief scientist ministry of Education Israel. The studies were conducted in accordance with the local legislation and institutional requirements. Written informed consent for participation in this study was provided by the participants’ legal guardians/next of kin.

## Author contributions

SS: Writing – original draft, Methodology, Investigation, Formal analysis, Conceptualization. OL: Writing – review & editing, Methodology, Conceptualization. DT-C: Writing – original draft, Methodology. AB: Writing – review & editing, Data curation. SD: Writing – review & editing, Data curation.
